# Exploring Built-Up Indices and Machine Learning Regressions for Multi-Temporal Building Density Monitoring Based on Landsat Series

**DOI:** 10.3390/s22134716

**Published:** 2022-06-22

**Authors:** R Suharyadi, Deha Agus Umarhadi, Disyacitta Awanda, Wirastuti Widyatmanti

**Affiliations:** Department of Geographic Information Science, Faculty of Geography, Universitas Gadjah Mada, Yogyakarta 55281, Indonesia; suharyadir@ugm.ac.id (R.S.); deha.agus.u@mail.ugm.ac.id (D.A.U.); disyacitta.awanda@mail.ugm.ac.id (D.A.)

**Keywords:** building density, Landsat, built-up indices, image transformations, regression

## Abstract

Uncontrolled built-up area expansion and building densification could bring some detrimental problems in social and economic aspects such as social inequality, urban heat islands, and disturbance in urban environments. This study monitored multi-decadal building density (1991–2019) in the Yogyakarta urban area, Indonesia consisting of two stages, i.e., built-up area classification and building density estimation, therefore, both built-up expansion and the densification were quantified. Multi sensors of the Landsat series including Landsat 5, 7, and 8 were utilized with some prior corrections to harmonize the reflectance values. A support vector machine (SVM) classifier was used to distinguish between built-up and non built-up areas. Regression algorithms, i.e., linear regression (LR), support vector regression (SVR), and random forest regression (RFR) were explored to obtain the best model to estimate building density using the inputs of built-up indices: Urban Index (UI), Normalized Difference Built-up Index (NDBI), Index-based Built-up Index (IBI), and NIR-based built-up index based on the red (VrNIR-BI) and green band (VgNIR-BI). The best models were revealed by SVR with the inputs of UI-NDBI-IBI and LR with a single predictor of UI, for Landsat 8 (2013–2019) and Landsat 5/7 (1991–2009), respectively, using separate training samples. We found that machine learning regressions (SVM and RF) could perform best when the sample size is abundant, whereas LR could predict better for a limited sample size if a linear positive relationship was identified between the predictor(s) and building density. We conclude that expansion in the study area occurred first, followed by rapid building development in the subsequent years leading to an increase in building density.

## 1. Introduction

The urban category is one of the important subjects for achieving sustainable development goals [1]. The studies in urban categories cover various phenomena including physical urban or urban morphology, urban communities, job availability, biodiversity, economic, transportation, and the urban environment [2,3,4]. Urban development is required to understand and mitigate the possible issues and challenges regarding its outward expansion. Vast urban area expansion has a trade-off for people and the environment. Based on socio-economic perspectives, urban areas provide benefits to support economic activities, but on the other hand, the areas potentially become prone to social conflicts such as social inequality and criminal events [5]. In addition, the environment also could be degraded especially in the suburban and rural areas, even in the urban area itself [6,7,8]. The increase in built-up areas costs agriculture and natural space that may affect environmental health [9]. It is also related to the increase in population and the demand for infrastructure development caused by built-up area expansion. The definition of urban is quite complex depending on the perspectives. It could be defined based on population density, or physical aspects such as building density [4,10]. Therefore, the building density information becomes one of the urban indicators to understand urban development.

Previous studies on building density showed its relationship to other phenomena such as macroclimate, urban heat island (UHI), energy use efficiency, social interaction, urban environment, and economy [5,11,12,13,14,15,16]. Building density is one of the variables that influences the variation of the urban micro-climate [17]. Building density also affects the UHI, where the higher building density increases the UHI intensity [18,19]. On the other hand, low building density with plenty of green spaces decreases the UHI intensity [12,20]. In another case, energy use efficiency was able to be examined from building density information, and some studies found that compact or dense built-up areas reduced energy consumption depending on technological advancement [13,14]. Physical interaction is inevitable in a densely built-up area, which increases the potential for crime, disease transmission, and social needs [15,21]. In addition, high building density is also related to population growth, economic productivity, land prices, transportation services, infrastructure, and the number of vacancies [5,22]. Based on previous research, building density information is necessary, especially the building density data with spatial and temporal information for a comprehensive urban study [4].

Globally, urban areas have been rapidly growing in recent years [23,24]. A multi-scale and multi-temporal analysis is required to understand the urban development patterns. The traditional methods to collect information on urban parameters are outdated and cost a lot of resources. Remotely sensed data are the key to providing convenient approaches for collecting and analyzing urban development [4,25]. The immense number of available remote sensing data in the various specifications allows the extraction of urban-related information more efficiently. For instance, the well-known and free remote sensing data, Landsat, has been providing high-quality images with moderate to high resolution for several decades [26,27]. These circumstances help urban scientists to tackle spatial and temporal challenges [4]. Multi-scale and multi-temporal urban research are benefitted due to remote sensing data availability, for example, multi-temporal urban growth monitoring derived from remote sensing data such as Landsat satellites [28,29,30,31]. Furthermore, the advancement of earth observation satellite sensors to retrieve thermal information such as MODIS and Landsat are useful for UHI monitoring [16,32,33,34]. The multispectral sensor also enables built-up area mapping using spectral transformation approaches [35,36].

Remote sensing-based urban studies mostly utilized built-up indices that were provided by previous studies, including Urban Index (UI) [37], Normalized Difference Built-up Index (NDBI) [38], Index-based Built-up Index (IBI) [39], and NIR-based built-up index using the red band (VrNIR-BI) and using the green band (VgNIR-BI) [40]. Kawamura et al. [37] developed UI by using NIR and mid-infrared (MIR) to distinguish built-up areas through satellite images proven by a strong positive relationship with urban density. Zha et al. [38] proposed NDBI using bands 4 and 5 considering the contrast reflectances in built-up features yet with a slight difference in other objects. Both UI and NDBI share the same possible issue in the similar values between built-up and bare land, although they are capable of separating between built-up and vegetation features. Therefore, Xu [39] developed IBI by integrating NDBI with the Soil-adjusted Vegetation Index (SAVI) and the Normalized Difference Water Index (MNDWI) to improve built-up area extraction thus the background noise of built-up areas can be suppressed. On the other hand, Estoque et al. [40] proposed VrNIR-BI and VgNIR-BI by utilizing visible spectral bands (green and red) and NIR to distinguish built-up areas from dry vegetation and grassland. Bhatti et al. [41] modified NDBI by integrating the temperature data and other indices such as the Normalized Difference Vegetation Index (NDVI) and the MNDWI, which showed significant improvement in distinguishing vegetation and water features yet still struggled to separate the built-up area from bare soil.

Built-up area extraction is well studied [35,36,42,43,44,45,46], however, the study of building density using remote sensing data is still limited where most of the studies exploited the use of high-resolution images [10,47,48]. Built-up density has been more widely studied than building density by extracting the built-up area and calculating the density based on a given mapping unit, commonly using the administration boundary [49,50,51]. For instance, a study by Shahfahad et al. [49] used the IBI to obtain the built-up area which was generated to a built-up density for each municipality. In the case of building density, studies by [52,53] directly used the values of the NDBI to represent the building density. However, this method caused misleading information since an empirical/semi-empirical statistical approach is required to estimate the building density from the transformation index. A linear regression using a combination of indices (NDBI, SAVI, NDWI, and thermal band) was performed to model building density percent, which showed a comparable result [54]. Another study explored several indices for multi-temporal building density estimation, showing that the VgNIR-BI outperformed other indices [55]. Along with the availability of historical Landsat data, the multi-decadal building density change is worth exploring.

On the other hand, the comparison of multiple regression algorithms such as linear and machine learning regression for building density has not been widely studied yet. The assessment of linear and machine learning regression was applied to construct a spatial model for specific applications [56,57]. The performance of a certain regression algorithm depends on the situation of the study [56]. Multiple regression methods need to be considered in constructing a particular model that can potentially achieve the most optimum results.

Investigations on the comparison of the developed indices are still limited, while the use of machine learning algorithms with the combination of built-up indices for multi-temporal building density estimation has not been adequately studied. To address this gap, this study aims to assess the best model for building density extraction based on previously mentioned built-up indices using multiple regression methods. Furthermore, this study also investigates multi-temporal building density information based on the multi-sensor of Landsat imageries. The best combination of regression analysis and built-up indices is expected to be achieved to provide better building density estimation and decadal built-up area expansion analysis.

## 2. Study Area

**Figure 1:**
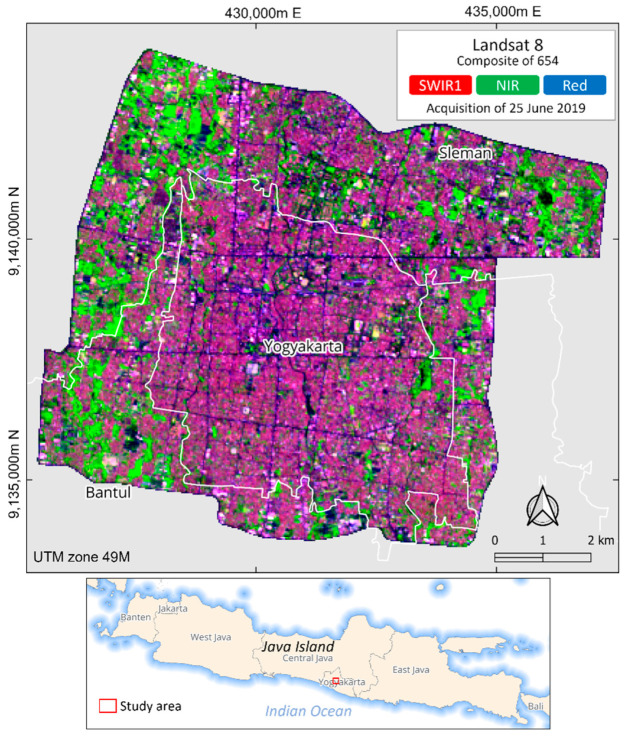
Study area with the background of Landsat 8 composite of 654. White lines indicate the administrative boundary at the regency level.

Yogyakarta City is located in the middle of the Province of the Special Region of Yogyakarta, a typical medium-sized and densely populated urban area of Java Island and one of the rapidly growing cities in Indonesia. The high growth rate of urbanization in urban areas during the past two decades has increased the community’s need for land. The population was concentrated in the middle of the city in the early 1990s, which then developed towards the north in the following decade, and spread to the suburban area around the city [58]. Considering the development in the periphery, our study covers the peri-urban areas using the boundary of a ring road encircling the city (Figure 1).

## 3. Materials and Methods

**Figure 2:**
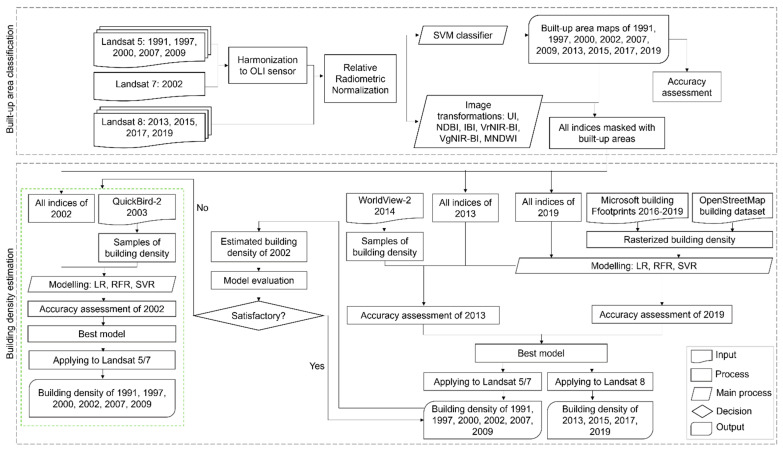
The flowchart of the study generally consists of built-up area classification and building density estimation.

This study utilized Landsat series images obtained from 1991 to 2019 as the primary datasets. Ground truth data of building density were derived from Microsoft building footprints, OpenStreetMap building dataset, and visual interpretation on QuickBird-2 and WorldView-2. In general, the methodology consists of built-up area classification and building density estimation as illustrated in Figure 2.

### 3.1. Landsat Data and Pre-Processing

Landsat provides open access to newly acquired imageries as well as the archives initiated with the launch of Landsat 1 in 1972 [31]. Thematic Mapper (TM) sensor carried onboard Landsat 5 offers the continuity of multispectral imaging passed on to Landsat 7 Enhanced Thematic Mapper Plus (ETM+) and Landsat 8 Operational Land Imager (OLI) at the same resolution of 30 m. Therefore, the Landsat series offers interannual earth observation time series analysis. Taking the advantage of the multi-decadal availability, we utilized the series of Landsat images from 1991 to 2019 in this study, consisting of Landsat 5 TM, Landsat 7 ETM+, and Landsat 8 OLI, as listed in Table 1.

Google Earth Engine cloud computing platform was used to obtain all images and perform the pre-processing. All Landsat images are at level 2 that were atmospherically corrected by the Landsat Ecosystem Disturbance Adaptive Processing System (LEDAPS) for Landsat 5 and 7 [59] and Land Surface Reflectance Code (LaSRC) for Landsat 8 [60]. To minimize the bias of the images, clouds and cloud shadows were removed by using QA_PIXEL band generated from the CFMask algorithm [61].

Compared to its predecessors, Landsat 8 has improved the sensor specifications such as the spectral wavelength ranges and radiometric resolution, thus harmonization between the sensors is encouraged for time series analysis. We applied the equations from Roy et al. [62] to harmonize both Landsat 5 and Landsat 7 with Landsat 8 for the bands of Blue, Green, Red, Near-Infrared (NIR), Shortwave Infrared-1 (SWIR-1), and Shortwave Infrared-2 (SWIR-2). The equations are as follows:

(1) $$\rho Blue_{harmonized}=0.0003+0.8474\;\rho Blue$$

(2) $$\rho Green_{harmonized}=0.0088+0.8483\;\rho Green$$

(3) $$\rho Red_{harmonized}=0.0061+0.9047\;\rho Red$$

(4) $$\rho NIR_{harmonized}=0.0412+0.8462\;\rho NIR$$

(5) $$\rho SWIR1_{harmonized}=0.0254+0.8937\;\rho SWIR1$$

(6) $$\rho SWIR2_{harmonized}=0.0172+0.9071\;\rho SWIR2$$

where ρ is the reflectance value of the corresponding bands.

After harmonization was applied, another correction was also performed, namely relative radiometric normalization (RRN) to further harmonize the values [63]. RRN was employed by applying a linear regression for each band between the reference and subject images [64]. The harmonized images of 1991, 1997, 2000, 2002, 2007, 2013, 2015, 2017, and 2019 were corrected using the single reference of the image of 2009 considering its temporal position in the middle of the data used. Pseudo-invariant features (PIFs) for the normalization were selected on the man-made objects (e.g., airports and big buildings) and deep water bodies (e.g., sea surface and lakes) which are deemed to have stable spectral values over observation time [65]. A linear regression was constructed separately for each band of particular images taking the reference (image of 2009) as the independent variable. All the regression analyses are significant (*p*-value < 0.05) with the report presented in Table A1 in Appendix A.

### 3.2. Building Datasets

We used building datasets derived from several sources for modeling and accuracy assessment as listed in Table 2. The latest building dataset was obtained from Microsoft building footprints and OpenStreetMap. Microsoft released open datasets of building footprints in the Philippines, Indonesia, and Malaysia generated from Maxar imagery acquired in 2016–2020 [66]. The pixels of building objects were identified using deep neural networks (DNNs) semantic segmentation and then were converted into polygons [67]. The results were evaluated showing the precision and recall of 88.64% and 77.53%, respectively, across the three nations [66]. As reported and observed visually in the study area, some buildings in the dense urban area were not identified. Therefore, we combined it with building data from OpenStreetMap generated from visual digitization. To match with satellite imagery used, the building datasets were then rasterized at a resolution of 30 m.

In addition, two other building density datasets were retrieved from the WorldView-2 image acquired on 19 August 2014 and a mosaic of QuickBird-2 image dated in 2003, with a total of 50 and 270 samples, respectively. Building objects were firstly delineated visually based on the grid samples of Landsat image, then converted into raster format at the same resolution as Landsat.

### 3.3. Built-Up Area Classification

A binary supervised classification was applied for each observation time to distinguish the built-up area from other land covers using Dzetsaka plugin in QGIS [68]. The algorithm used is Support Vector Machine (SVM) classifier considering the neglect of the data distribution and the suitability of the limited training data and high-dimensional inputs [69]. This algorithm aims to find a hyperplane that discriminates the training samples according to the assigned classes [70]. Only two samples were used in the classification, i.e., built-up and non built-up areas. Learning methods were performed separately by taking different training samples for each corresponding imagery. Training samples were selected with the help of the high-resolution Google Earth images and the local knowledge of interpreters. The accuracy of each result was assessed by confusion matrices with the same samples across the observation time to evaluate the classification.

### 3.4. Building Density Extraction

The extraction of building density used the inputs of image transformations, consisting of Urban Index (UI), Normalized Difference Built-up Index (NDBI), Index-based Built-up Index (IBI), and two visible-based indices combined with NIR band (i.e., VrNIR-BI and VgNIR-BI) as listed in Equations (7)–(11). UI was firstly developed by seeing the converse relationship between the reflectance of urban areas between NIR and SWIR spectrum [37]. The equation of NDBI was formulated by Zha et al. [38] using the SWIR-1 band, instead of SWIR-2 as used in UI. Xu [39] developed IBI considering three established indices, i.e., soil adjusted vegetation index (SAVI), modified normalized difference water index (MNDWI), and NDBI, for the rapid extraction of built-up objects. To minimize the bias in separating built-up lands from dry vegetation, two visible-based built-up indices (VrNIR-BI and VgNIR-BI) were constructed based on the red and green bands, separately [40].

(7) $$\mathrm{UI}=\frac{\rho SWIR2-\rho NIR}{\rho SWIR2+\rho NIR}$$

(8) $$\mathrm{NDBI}=\frac{\rho SWIR1-\rho NIR}{\rho SWIR1+\rho NIR}$$

(9) $$\mathrm{IBI}=\frac{\frac{2\rho SWIR1}{\rho SWIR1+\rho NIR}-\left(\frac{\rho NIR}{\rho Red+\rho NIR}+\frac{\rho Green}{\rho Green+\rho SWIR1}\right)}{\frac{2\rho SWIR1}{\rho SWIR1+\rho NIR}+\left(\frac{\rho NIR}{\rho Red+\rho NIR}+\frac{\rho Green}{\rho Green+\rho SWIR1}\right)}$$

(10) $$\mathrm{VrNIR}-\mathrm{BI}=\frac{\rho Red-\rho NIR}{\rho Red+\rho NIR}$$

(11) $$\mathrm{VgNIR}-\mathrm{BI}=\frac{\rho Green-\rho NIR}{\rho Green+\rho NIR}$$

Three regression algorithms were employed to estimate building density, i.e., linear regression, random forest regression, and support vector regression using Python-based EnMAP-Box in QGIS [71]. A linear regression algorithm is used considering the linear relationship between the built-up indices with the presence of building objects, shown by the higher values [37]. The linear model was built individually for each index. Two multiple linear regression models were then developed using the inputs of all indices and UI-NDBI-IBI combined.

Random forest regression (RFR) is a regression-based random forest learning algorithm that is one of the ensemble machine learning models. The prediction was created by multiple decision trees and aggregation on the trees in the forest that were bootstrapped [72]. The optimal hyperparameter was determined for the number of trees in the forest (ntrees = 100, 200, 500, 1000) within the RFR processing using a grid search with a 5-fold cross-validation.

SVM can solve the regression problems as well, namely support vector regression (SVR), with the output of continuous data [73]. The radial basis function (RBF), a non-linear kernel, was selected due to its capability to outperform other kernels [74]. Similar to RFR, hyperparameters were also optimized on the parameters of the gamma value (gamma = 0.1, 0.2, 0.5) and the regularization parameter (C = 1, 10, 100). For both RFR and SVR, the two sets of inputs are used in the learning, i.e., all indices and UI-NDBI-IBI combined, separately.

Firstly, the dataset of 2019 was used in the modeling using linear regression, RFR, and SVR (please see Figure 2). On the building density dataset derived from Microsoft and OpenStreetMap, we separated the samples into 70% for modeling and 30% for accuracy assessment. Root mean square error (RMSE) was calculated for assessing the validation score. Another method used for accuracy assessment is the upper range of accuracy using standard error of estimation with a 95% confidence level (CL) [75]. All models were then applied to the dataset of 2013, which were then assessed the accuracy using building density data obtained from WorldView-2 (2014). An evaluation was conducted based on the validation and accuracy assessment to find the best model.

The best model obtained was applied to all datasets of the observation period (1991–2019). Subsequently, evaluation was performed using the reference of the building density dataset obtained from QuickBird-2 data (2003) on the modeling result of Landsat 7 imagery (2002). If the evaluation is not satisfactory, remodeling will be performed separately for Landsat 5 and Landsat 7, considering both similar sensor characteristics (Figure 2).

## 4. Results

### 4.1. Transformation of the Built-Up Area

**Figure 3:**
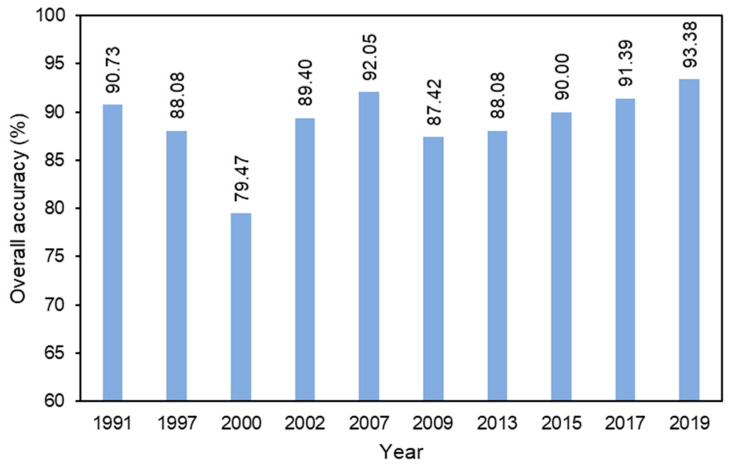
The overall accuracy of land use classifications.

**Figure 4:**
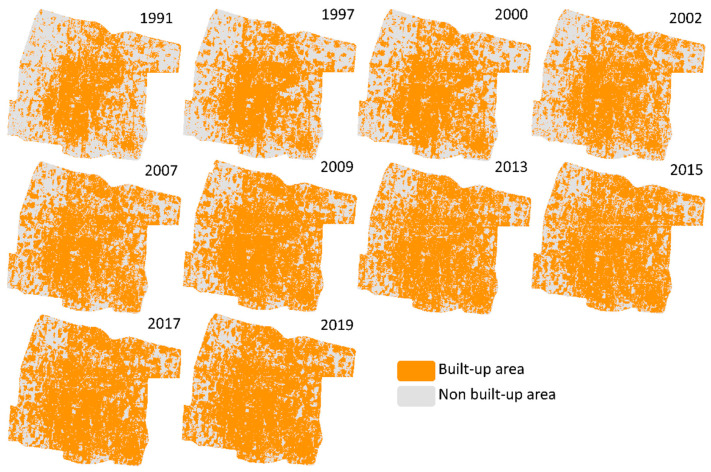
Land cover classification of built-up and non built-up area using SVM classifier based on Landsat images.

The SVM classification resulted in a good performance based on accuracy assessment by means of high-resolution images (Google Earth) and the local knowledge of interpreters for each date of the images. The results showed that the accuracy varied from 79.47% to 93.38% (Figure 3). Most of the overall accuracies reached more than 85% except for the classification based on Landsat 5 in 2000 (79.47%), in which several misclassifications occurred due to the weather conditions that subtly affect the quality of the images. However, the classifications on Landsat 5 and 7 in 1991, 1997, 2002, 2007, and 2009 performed satisfactorily with the accuracy of 90.73%, 88.08%, 89.04%, 92.05%, and 87.42%, respectively. On the other hand, the results of classification on Landsat 8 (2013–2019) were quite good with an accuracy of 88.08%, 90.00%, 91.39%, and 93.38%, respectively. The accuracy varied depending on the quality of the images. Nevertheless, the results visually represented the distribution of the built-up area quite well (Figure 4) and are reliable enough.

The built-up area classification based on the SVM classifier showed satisfactory results. Although Landsat 8 has slightly better sensor specifications than Landsat 5 and 7, their performances in classifying built-up and non built-up areas are quite similar based on the accuracy assessment. It should be noted that these results were obtained after the rigorous calibration and harmonization of Landsat sensors were carried out. The SVM classifier itself already outperformed other widely used classification methods such as maximum likelihood, as reported by previous studies [76,77]. However, the training data have an important impact on SVM classification. Therefore, the sample optimization of training data for SVM classification was carefully selected to achieve the best performance, including pure pixel, sample size, and sample in a homogeneous area [77,78,79,80].

The lowest accuracy (79.47%) in 2000 was due to shadows of high clouds covering the small area in the south-eastern part of the image scene causing lower reflectance values on the area. Therefore, SVM failed to classify some building objects, yet still performed reasonably. The low quality of the image caused noise and ambiguous spectral responses that complicate the SVM algorithm to distinguish between two classes. On the other hand, the overall misclassification occurred also due to a similar spectral response between built-up areas and bare soil, especially dry soil [81,82]. A wide area of impervious surfaces such as parking lots, which are categorized as a non built-up areas, also caused an error [83]. The different number of training sample and distribution in each image affected the performance as well. Training sample selection adjusted to the condition and land-use change dynamic causes the accuracy to vary in each image. However, the performance of the SVM classification showed satisfactory results with most of the accuracies being more than 85%.

As shown in Figure 4, the built-up area in Yogyakarta increased and spread rapidly from the year 1991 to 2019. The distribution of the built-up changes was observed nearly equally in all directions, with extensive changes occurring in the north, east, and southeast parts. In 1991, the built-up area was concentrated in the middle part of the study area, yet in the period between 1997 and 2002, it started to increase in the northeast and southeast directions. The built-up area in the western part started to develop and, in the east part, intensively increased from 2007 until 2015. There was a slight change observed in 2015 and 2019, however, the built-up area has been increasing during these two years and most of the study area was covered by the built-up area in almost all directions.

**Figure 5:**
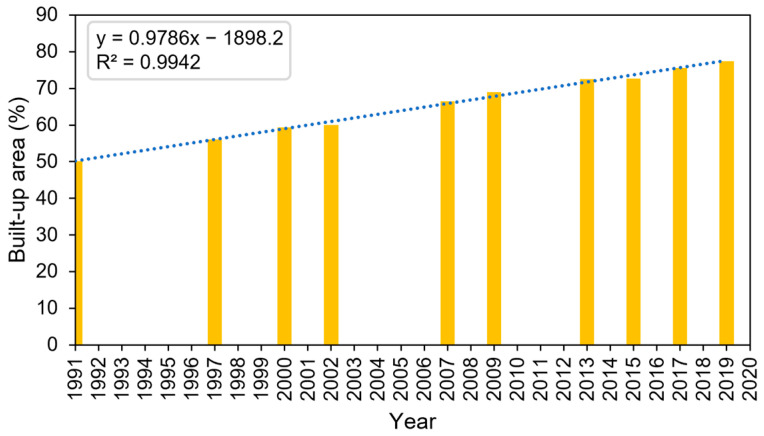
Percentage of built-up areas classified from Landsat images with a linear fit line showing a positive trend.

Based on Figure 5, the built-up area has increased up to 27.35% of the study area (2250 ha) from 1991 to 2019. In general, consistent increases in the built-up area were identified throughout the observation periods, except in 2002 and 2013 when only a slight expansion was observed. Calculated by a linear fit, the acceleration of the built-up expansion is about 80 ha per year or 0.98% of the study area per year. Furthermore, this built-up area classification was used to mask the built-up area only for the building density model.

### 4.2. Building Density Estimation

Within RFR and SVR processing, GridSearchCV was involved to look for the best model based on the given parameters. For modeling based on Landsat 8 images, the RFR model with the 1000 ntrees was selected as the best parameter for both using all indices and UI-NDBI-IBI. Meanwhile, the best parameters of SVR are different for both predictor sets, i.e., C = 1 and gamma = 0.1 for all indices, and C = 100 and gamma = 0.1 for UI-NDBI-IBI.

**Figure 6:**
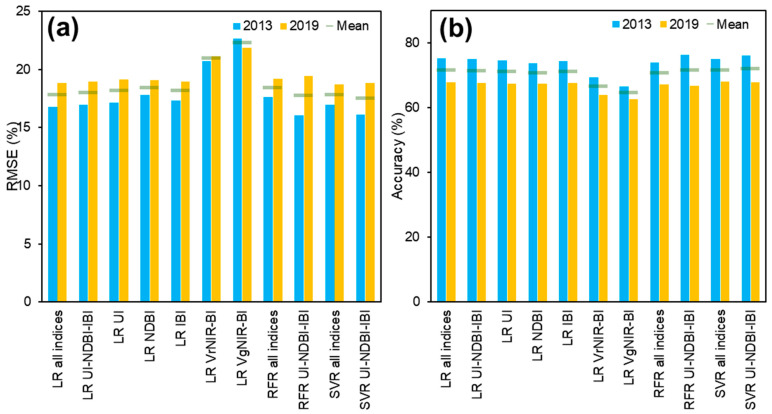
(**a**) RMSE and (**b**) upper range of accuracy at 95% confidence level for each building density estimation using linear regression (LR), random forest regression (RFR), and support vector regression (SVR), tested on the datasets of 2013 and 2019.

All learning methods were then applied for the images of 2013 and 2019. It is intended to assess the accuracy to evaluate the models for the multi-temporal consistency. The results showed that SVR with UI-NDBI-IBI predictors managed to have the lowest RMSE at 17.47% (mean RMSE calculation based on 2013 and 2019), and was also confirmed with the highest upper range accuracy at 72.07% (Figure 6). Other methods with multiple inputs had the mean RMSE values of less than 18%, i.e., RFR UI-NDBI-IBI (RMSE = 17.76%, accuracy = 71.57%), LR all indices (RMSE = 17.82%, accuracy = 71.56%), SVR all indices (RMSE = 17.83%, accuracy = 71.56%), and LR UI-NDBI-IBI (RMSE = 17.96%, accuracy = 71.34%), except RFR all indices (RMSE = 18.42%, accuracy = 70.63%). Both visible indices (VrNIR-BI and VgNIR-BI) possessed mean RMSE and accuracy values of above 20% and below 70%, respectively.

**Figure 7:**
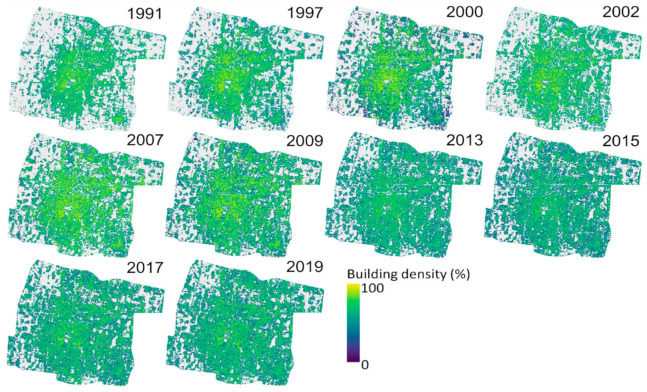
Building density maps estimated by support vector regression (SVR). Overestimates are shown in the images of 1991–2009 derived from Landsat 5/7.

**Figure 8:**
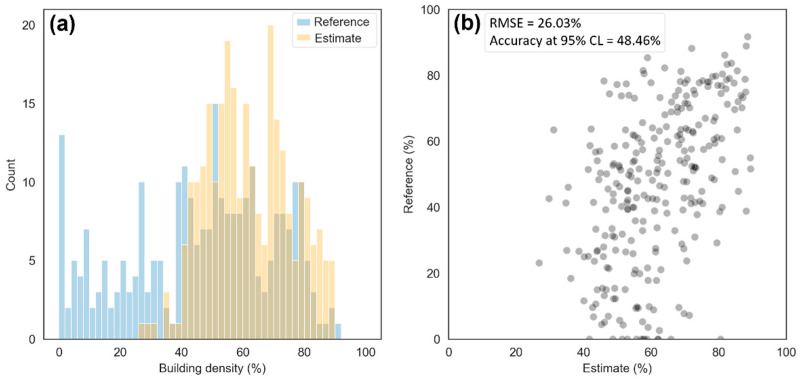
Comparison of the distribution between reference data and estimated building density in the image of 2002, plotted as (**a**) histograms and (**b**) a scatterplot.

The best model, SVR UI-NDBI-IBI, was then applied to all datasets. As shown in Figure 7, extreme higher density values were detected visually in the center of the study area on images of 1991–2009 that are derived from Landsat 5/7, compared to those in 2013–2019. To validate the overestimate, the estimated building density of 2002 was analyzed with 270 reference data (delineated building density from QuickBird-2 2003). Figure 8 shows the histograms and a scatterplot, apparently showing that the estimated values (mean = 61.85%) are extremely higher than the reference (mean = 46.61%). In addition, the RMSE value indicated a high error at 26.03% with a poor upper range of accuracy (48.46%).

Since the model was not satisfactorily applied for Landsat 5/7, we remodeled the building density using the training samples of 2003 applied on Landsat 7 acquired in 2002. Samples were split, i.e., 220 and 50 samples for building the model and testing, respectively. As the modeling used different reference data, the best parameters were also assessed separately. The best RFR models are with 500 and 200 ntrees for the inputs of all indices and UI-NDBI-IBI, respectively. For SVR, the best parameters for modeling are C = 1 and gamma = 0.2 for all indices, and C = 10 and gamma = 0.1 for UI-NDBI-IBI.

**Figure 9:**
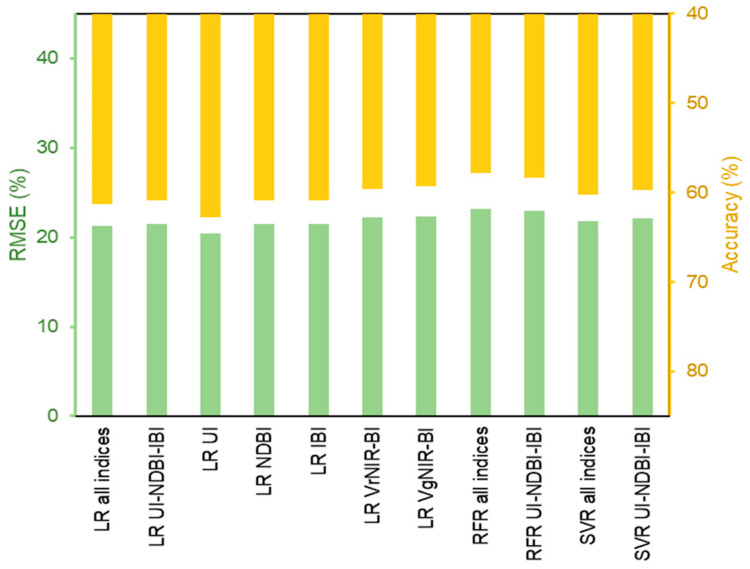
Values of RMSE and upper range of accuracy at 95% confidence level for building density estimation using linear regression (LR), random forest regression (RFR), and support vector regression (SVR) on Landsat 7 data.

In contrast to Landsat 8, the high accuracy models using Landsat 7 are dominated by LR methods (Figure 9), with the best model using the predictor of UI (RMSE = 20.48%, accuracy = 62.76%), followed by all indices (RMSE = 21.32%, accuracy = 61.25%), NDBI (RMSE = 21.52%, accuracy = 60.85%), UI-NDBI-IBI (RMSE = 21.54%, accuracy = 60.84%), and IBI (RMSE = 21.54% accuracy = 60.84%). The least accurate models are RFR using both predictor sets, i.e., all indices (RMSE = 22.93%, accuracy = 58.31%) and UI-NDBI-IBI (RMSE = 23.18%, accuracy = 57.86%). The best model was then applied to the datasets of 1991-2009 considering the similar sensor characteristics designed in Landsat 5 and 7.

**Figure 10:**
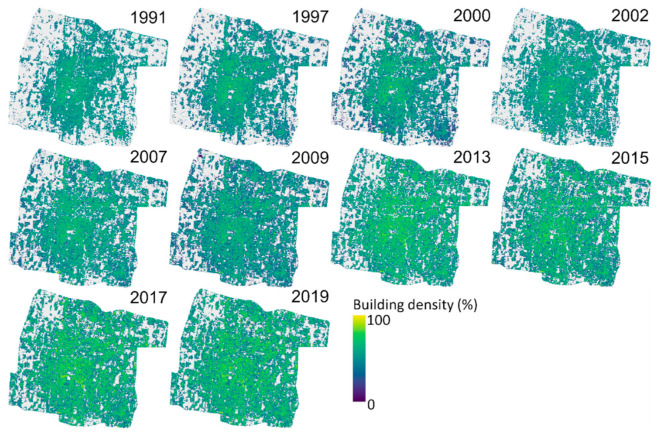
Final building density maps derived from linear regression on Landsat 5/7 (1991–2009) and support vector machine on Landsat 8 images (2013–2019).

The best models derived from the accuracy assessment were then used to estimate building density. SVR was applied for the UI-NDBI-IBI images of 2013, 2015, 2017, and 2019, whereas LR was performed for UI images of 1991, 1997, 2000, 2002, 2007, and 2009 as shown in Figure 10. Visually, the higher density is identified from 1991 to 2002 in the center of the city, which is gradually getting less dense towards the outskirts. The high building density started to spread afterward until it is nearly equally distributed in the study area in 2019.

**Figure 11:**
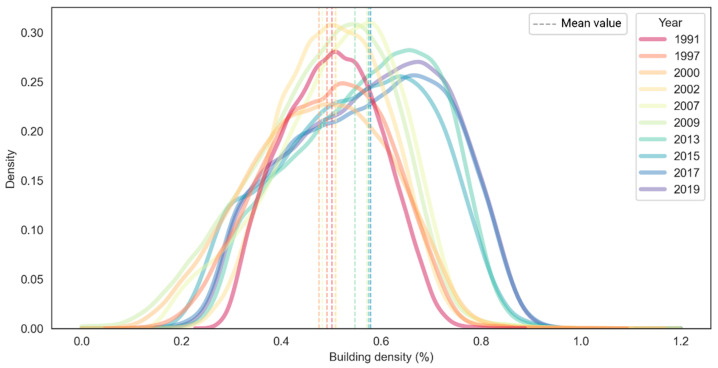
Multi-temporal data distribution of building density map, plotted using kernel density estimation (kde).

All pixels of building density images were then extracted to plot them into kernel density estimation (kde) in order to see the trends (Figure 11). The kde plot reveals a positive trend of building density that, in general, shows the building of built-up areas in the study area is getting denser over time. As visually observed, the kde plot was normally distributed in 1991, yet the graphs become skewed to the left, and the skewness is more obvious in the last four observation periods (2013–2019). This corresponds to the quantified mean values, rising from 50.17% in 1991 to 51.03% in 2007 and 57.88% in 2019.

**Figure 12:**
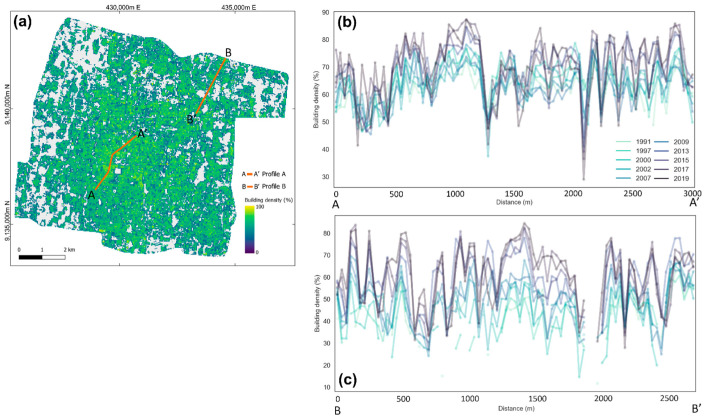
(**a**) Profile lines with the background of estimated building density in 2019 and (**b**,**c**) the profiles A and B of multi-temporal building density.

To see the changes in building density multi-temporally, we plotted two profile lines in the study area, i.e., Profile A and Profile B, representing the development in the center of the city and the outskirts, respectively (Figure 12). It can be seen that the building density is getting higher in both profile lines. The growth in the periphery of the city is more significant indicated by the obvious increases in the last observation periods. It is due to the existence of dense built-up areas in the middle of Yogyakarta City observed in 1991. The negative extreme spikes in the profiles are the presence of roads and/or other objects with a small coverage of buildings.

## 5. Discussion

### 5.1. Multiple Landsat Sensors for Decadal Modeling

We demonstrated that the modeling applied by Landsat 8 trained in 2019 could achieve similar accuracy when tested in 2013. On the other hand, it was unable to perform well on Landsat 5/7, indicated by the overestimates in the images of 1991–2009 and a low accuracy calculated based on Landsat 7 of 2002, even though sensor harmonization and RRN were applied in the pre-processing method. This might be since the simple RRN used could not perfectly normalize the values between different sensors despite the same Landsat series. Ground reference data or PIFs in this study were selected manually. Although careful selection was carried out on the bright and dark subjects, this human intervention method could lead to subjectivity [65]. Several automatic PIF selections were developed and should be carried out to minimize the imprecise correction [84,85]. Machine learning-based non-parametric RRN methods, such as artificial neural networks (ANN) and SVM, can be potentially useful for more accurately normalizing images [86,87]. Nevertheless, our pre-processing methods could achieve good results for the same sensors. This would suggest that when different sensors are involved in time series analysis, a more rigorous correction is highly recommended.

### 5.2. Comparison of Regression Algorithms

Three regression algorithms were evaluated based on the RMSE and upper range accuracy values on the models using Landsat 8 and Landsat 7, separately. SVR outperformed other methods using Landsat 8, conversely, the non-parametric models (SVR and RFR) were less accurate than LR applied using Landsat 7. It is mainly due to the difference in sample size included in the modeling. Microsoft and OpenStreetMap building footprints provided the spatial data of the whole study area that were used for modeling on Landsat 8 data. This comprises 68,849 pixels, where 70% (48,194 pixels) of those are used in the model training. In contrast, the modeling on Landsat 7 used the 220 samples obtained by visual interpretation on Quickbird image. RFR is sensitive to the sample size that is benefited by large sample sizes to fit the model [88,89]. Thus, this algorithm is more capable of handling an enormous number of samples. In the case of modeling on Landsat 7, the number of samples was just enough to be used, thus SVR and RFR could not perfectly estimate the unexplained values. The prediction was better achieved by LR since a linear positive relationship was possessed between the built-up indices as the predictors and the building density [37,54]. This confirms previous studies that LR has proved its capability to predict time series biophysical parameters although a limited sample size is used if there is a linear relationship between dependent and independent variables [90,91].

SVR using the inputs of UI-NDBI-IBI produced the highest accuracy among the others (Figure 6). The use of all indices in data training might lead to a less accurate model, as we observed that VrNIR-BI and VgNIR-BI models using the LR algorithm showed high RMSE values. It emphasized the need for reducing high-dimensional datasets to only involve the important inputs [89,92]. Meanwhile, UI employing the LR method outperformed the modeling using Landsat 7 data. This index has proved its capability of discriminating built-up areas [45] and also is capable of estimating building density in an urban area [37]. Yet, a study investigating the consistency of the best methods (UI with LR as well as UI-NDBI-IBI with SVR) applied in other urban regions needs to be addressed in the future.

### 5.3. Expansion and Densification of Built-Up Area

**Figure 13:**
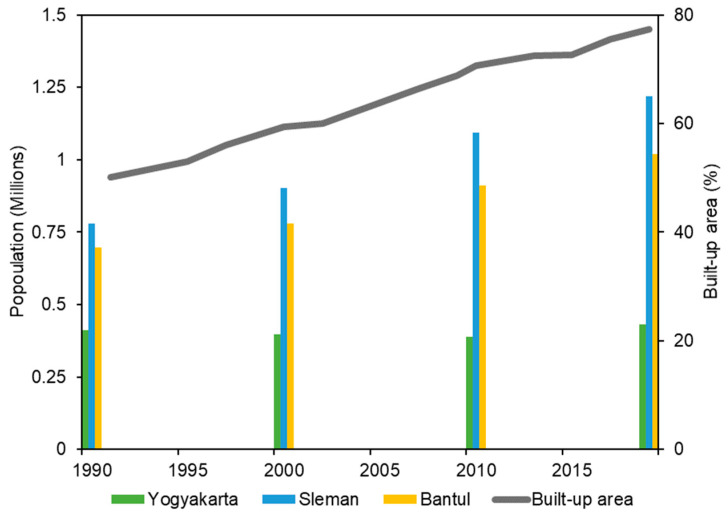
Growth of population density in Yogyakarta, Sleman, and Bantul compared to the expansion of the built-up area. Population data were adapted from Badan Pusat Statistik [103] and Kuncoro [102].

The results of SVM classification from 1991 to 2019 clearly showed the expansion and densification of built-up areas. The urban area was normally growing and spreading year by year and it is related to human activities and their needs as well as population growth. It means that the built-up area development could be indicated by the rising number of inhabitants [93]. Based on the population data, our study found that there is a positive relationship between the increase in a built-up area and population growth (Figure 13). The population has rapidly increased in the Sleman and Bantul regencies that surround the city center, however, Yogyakarta City was quite stable throughout the periods studied. Based on the distribution of the increase in built-up land, the most intensive changes occurred in areas adjacent to the Yogyakarta City boundary. This indicates that the distribution of population growth occurs in areas adjacent to the administrative boundaries of the city of Yogyakarta. This finding is matched with the built-up area changes from the SVM classification.

The direction of the urban growth usually follows the development of economic activities that could be recognized by an increase in the built-up area [94,95]. It is indicated by the rapid development of infrastructure, facilities, and settlements, with the purpose of decentralized economic activity. These situations attracted people from outside the city to develop and integrate the infrastructure and facilities, therefore, it caused urban morphology in the outer city to expand or, in other terms, agglomeration occurs [96,97]. In this case, the increase in economic activity in Yogyakarta due to the development of the tourism industry from 1991 to 2019 led to extensive infrastructure development as well as an increased built-up area [98,99,100]. Besides population growth and economic activity, the expansion was highly influenced by the presence of universities and schools of higher education that are mostly located in the northern part of the study area [101]. The increased availability of educational facilities has encouraged people from other cities to move for a better education. Furthermore, our study also found that agglomeration occurred in Yogyakarta City and the regencies around especially the Sleman and Bantul regions, the same as a previous study [102]. Future studies should involve infrastructure development to spatially understand the impacts on the expansion and densification of built-up areas.

The factors contributing to building densification are relatively more complex when compared to the expansion process. Areas that have undergone expansion will in turn be followed by building densification. Densification in urban areas is directly affected by the increased rate of urbanization and indirectly affected by the increase in community welfare [104]. The high rate of built-up area expansion as well as the densification in the late 2000s was due to the economic recovery after the national economic crisis in 1998 [105,106]. Previous studies reported that built-up areas were mostly converted from croplands in the study area [107,108,109]. Although the government has strengthened the policy regarding the croplands development to secure food sustainability [110,111], a positive trend in built-up areas was still detected and might be rising in the future.

## 6. Conclusions

This study demonstrates the extraction of building density in Yogyakarta through two phases, i.e., built-up area classification and building density estimation, of which the first-mentioned results were also used as a mask to avoid bias in modeling building density. Multi-temporal modeling of building density is still challenging for the series of Landsat data due to different sensor specifications. Although sensor harmonization and relative radiometric normalization (RRN) were applied, the model trained based on Landsat 8 in 2019 was unable to accurately predict using Landsat 5/7 images, resulting in overestimates. Using training samples of 70% of the total area based on Landsat 8 (2019), the support vector machine (SVR) algorithm using the inputs of Urban Index, Normalized Difference Built-up Index, and Index-based Built-up Index (UI-NDBI-IBI) performed the best, tested by accuracy assessment in 2019 and 2013 (mean RMSE = 17.47%, accuracy = 72.07%). On the other hand, the best model processed using Landsat 7 (2002) was achieved by linear regression (LR) with a single input of UI (RMSE = 20.48%). This is because the limited samples used in modeling (220 samples) can be better trained by LR compared to non-parametric algorithms. Our results conclude that built-up area expansion, as well as densification in the study area, occurred in the last three decades (1991–2019) with a linear positive trend. Expansion occurred first which was subsequently followed by rapid building construction observed by the rise in building density. The population growth in the vicinity of the city, i.e., fringe areas, has a great impact on both the built-up area development and the densification.

## Figures and Tables

**Table 1:** List of Landsat images used in this study.

No.	Acquisition Date	Sensor	No.	Acquisition Date	Sensor
1	31 August 1991	Landsat 5 TM	6	31 July 2009	Landsat 5 TM
2	25 April 1997	Landsat 5 TM	7	24 June 2013	Landsat 8 OLI
3	4 June 2000	Landsat 5 TM	8	14 June 2015	Landsat 8 OLI
4	21 August 2002	Landsat 7 ETM+	9	18 May 2017	Landsat 8 OLI
5	26 July 2007	Landsat 5 TM	10	25 June 2019	Landsat 8 OLI

**Table 2:** Sources of building datasets used in this study.

No.	Dataset	Processing	Purpose
1	Microsoft building footprints	Merging both datasets and rasterization	Model training and accuracy assessment of Landsat 8 of 2019
2	OpenStreetMap building		
3	WorldView-2 of 2014	Visual interpretation of building objects	Accuracy assessment of Landsat 8 of 2013
4	QuickBird-2 of 2003	Visual interpretation of building objects	Best model evaluation of Landsat 7 of 2002, and model training and accuracy assessment of Landsat 7 of 2002

## Data Availability

Processed data are available upon request.

## Appendix A

**Table A1:** Coefficient of determination (R^2^) values for the selection of PIFs between Landsat 5 dated in 2009 as the reference and the corresponding datasets. All values are significant (*p*-value < 0.05).

No.	Dataset	Bands					
		Blue	Green	Red	NIR	SWIR1	SWIR2
1	Landsat 5—1991	0.8039	0.8163	0.8185	0.8531	0.8547	0.8679
2	Landsat 5—1997	0.8854	0.8230	0.8675	0.8322	0.9127	0.9084
3	Landsat 5—2000	0.8952	0.8766	0.8944	0.8817	0.9323	0.9207
4	Landsat 7—2002	0.8080	0.8236	0.8046	0.8752	0.8693	0.8890
5	Landsat 5—2007	0.9516	0.9629	0.9399	0.9500	0.9169	0.9231
6	Landsat 8—2013	0.6901	0.7554	0.8000	0.8578	0.6905	0.6865
7	Landsat 8—2015	0.8658	0.9496	0.9329	0.9345	0.8694	0.8501
8	Landsat 8—2017	0.8816	0.9386	0.9328	0.9447	0.8895	0.8668
9	Landsat 8—2019	0.8732	0.9567	0.9329	0.9438	0.9115	0.8790
